# Understanding the Mechanisms by Which Epigenetic Modifiers Avert Therapy Resistance in Cancer

**DOI:** 10.3389/fonc.2020.00992

**Published:** 2020-06-24

**Authors:** Anthony Quagliano, Anilkumar Gopalakrishnapillai, Sonali P. Barwe

**Affiliations:** ^1^Nemours/Alfred I. duPont Hospital for Children, Wilmington, DE, United States; ^2^Department of Biological Sciences, University of Delaware, Newark, DE, United States

**Keywords:** epigenetic aberrations, chemoresistance, mechanism, cancer, epigenetic drugs, epigenetic combination therapies

## Abstract

The development of resistance to anti-cancer therapeutics remains one of the core issues preventing the improvement of survival rates in cancer. Therapy resistance can arise in a multitude of ways, including the accumulation of epigenetic alterations in cancer cells. By remodeling DNA methylation patterns or modifying histone proteins during oncogenesis, cancer cells reorient their epigenomic landscapes in order to aggressively resist anti-cancer therapy. To combat these chemoresistant effects, epigenetic modifiers such as DNA hypomethylating agents, histone deacetylase inhibitors, histone demethylase inhibitors, along with others have been used. While these modifiers have achieved moderate success when used either alone or in combination with one another, the most positive outcomes were achieved when they were used in conjunction with conventional anti-cancer therapies. Epigenome modifying drugs have succeeded in sensitizing cancer cells to anti-cancer therapy via a variety of mechanisms: disrupting pro-survival/anti-apoptotic signaling, restoring cell cycle control and preventing DNA damage repair, suppressing immune system evasion, regulating altered metabolism, disengaging pro-survival microenvironmental interactions and increasing protein expression for targeted therapies. In this review, we explore different mechanisms by which epigenetic modifiers induce sensitivity to anti-cancer therapies and encourage the further identification of the specific genes involved with sensitization to facilitate development of clinical trials.

## Epigenetics and Cancer

The term “epigenetics” refers to the study of heritable phenotypic changes that do not involve mutations in DNA sequence (1). These changes are centered around alterations in gene activity and expression; through a variety of processes including DNA methylation and histone modifications (2). DNA methylation is the covalent addition of a methyl group to the C-5 position of DNA cytosine rings by DNA methyltransferases. Gene promoter hypermethylation often results in transcription depletion leading to decreased gene expression (3). Conversely, hypomethylation of *ABCB1* promoter resulted in upregulation of ABCB1 protein and acquisition of taxane resistance via efficient drug efflux (4). In exceptional cases, promoter methylation of genes, like *TERT* gene encoding telomerase reverse transcriptase, leads to increased transcription and protein expression (5). Methylation in gene bodies also affects transcription; demethylation of gene bodies results in a decrease in gene transcription (6). These patterns of DNA methylation are retained during cell division and can persist across generations.

Histones are modified in multiple ways. These modifications alter chromatin structure and affect gene transcription by regulating the access of transcription machinery to DNA. For an excellent review on the different types of histone modifications, refer to Audia and Campbell (7). Enzymes that modify histone proteins also facilitate post-translational modifications in non-histone proteins, thereby affecting gene expression (8, 9). Acetylation of NFκB and methylation of tumor suppressor protein p53 promotes nuclear localization of these proteins and increases transcriptional activity of respective gene targets (10, 11). Due to the prominent role of these proteins in cancer progression and therapy resistance, targeting their post-translational modifications could have therapeutic benefit (12, 13).

**Figure 1:**
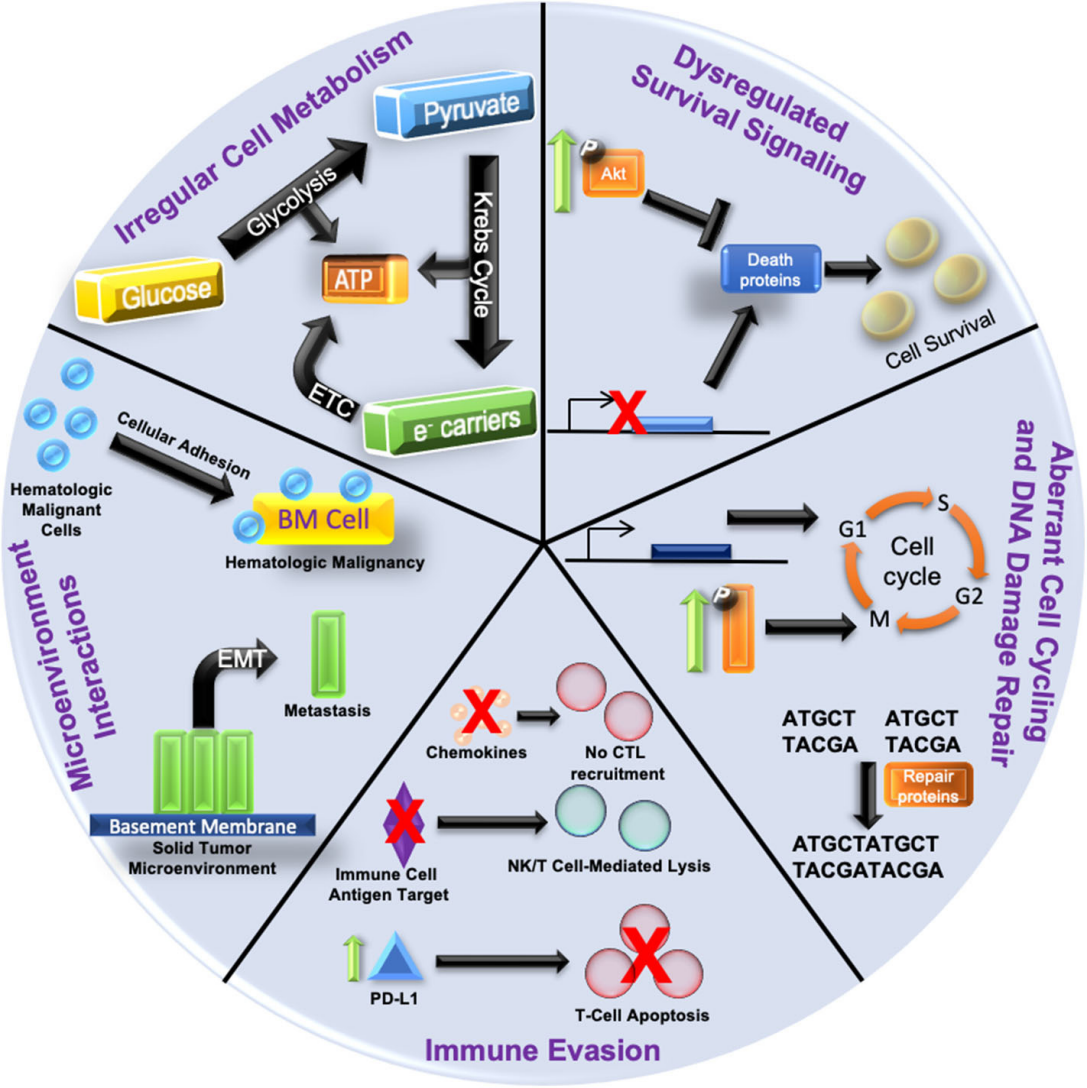
Hallmarks of Epigenetic Alteration-Induced Therapy Resistance Epigenetic dysregulation is a driving force in oncogenesis and the development of therapy resistance. (1) Increased pro-survival signaling (depicted by enhanced phosphorylation and activation of kinases such as Akt) can inhibit the expression of death proteins to promote cancer cell survival. The gene expression of death proteins (shown as transcriptional inhibition at the gene promoter) can also be disrupted, culminating in increased cell survival. (2) Aberrant cell cycling is caused by the over/under-expression of proliferative/checkpoint proteins (blue block with promoter), or increased activation of signaling pathways (shown by increased phosphorylation/activation) related to proliferation. DNA damage repair is augmented by an increased expression of repair proteins and disruption of checkpoint signaling. (3) Aberrant intracellular signaling can also alter cytokine expression and lead to reduced cytotoxic T lymphocyte (CTL) recruitment. Silencing of immune cell antigen targets can also suppress immune targeting of cancer cells (NK, natural killer). Increased expression of PD-L1 (green arrow and blue triangle) on cancer cells can augment the immune checkpoint response, resulting in T cell apoptosis. (4) Increased cellular adhesion within the bone marrow microenvironment (yellow block) in hematologic malignancies activates intracellular signaling pathways that protect malignant cells (blue spheres) from anti-cancer. Epithelial to mesenchymal transition (EMT) dislodges cancer cells (green blocks) from the solid tumor microenvironment and is the first step in metastasis. (5) Irregular cellular metabolism via overactive glucose metabolism leads to the Warburg effect favoring anabolic glycolysis over oxidative phosphorylation, and can render cells resistant to chemotherapeutics or antimetabolites. Resistance mechanisms are not restricted to just one of the categories; often with multiple categories being involved simultaneously.

Drastic alterations in the epigenetic landscapes occur in cancer cells (14). Aberrant epigenetic patterns function as key drivers in cancer initiation and progression; often a result of the silencing of tumor suppressor genes or induced overexpression of oncogenes (15). Several tumor suppressors, such as RASSF1A and CASP8, are frequently inactivated in multiple cancer subtypes via epigenetic downregulation rather than by genetic mutation. For an excellent review on this specific function in oncogenesis, see Kazanets et al. (16). On the other hand, certain oncogenes, such as c-Myc and insulin-like growth factor receptor 2 (IGF-2) are upregulated by epigenetic mechanisms (17). These epigenetic changes result in global dysregulation of gene expression; thereby solidifying the development of disease states (18). Anomalous epigenetic alterations can also lead to the acquisition of therapy resistance (19, 20). Figure 1 outlines how epigenetic-induced gene expression changes can give rise to multiple mechanisms of therapy resistance.

Efforts to revert these epigenetic changes via the use of epigenome modifying drugs have achieved some success, specifically when used in conjunction with other therapies. While these modifiers are “non-specific” in that they affect gene expression on a global level, their action elicits “specific” effects in malignant cells. This is due to the altered epigenome that is acquired during oncogenesis, highlighted by expression changes in tumor suppressor genes (silenced) and oncogenes (augmented) that are responsible for cancer progression or therapy resistance. Thereby, treatment with epigenetic drugs elicits a “specific” effect on cancerous cells by reverting these unique expression changes. Additionally, sensitivity to epigenetic modifiers can be genomic loci specific, possibly due in part to the three-dimensional chromatin structure (21–23). Thus, epigenetic modifiers possess the unique ability to be effective in a broad category of patients; albeit via altering the expression of a set of genes in a patient- specific manner (24).

In order to better understand the uses and indications of epigenetic modifiers in these combinations, it is necessary to uncover mechanisms of epigenetic drug-induced sensitization to anti-cancer therapy. Below, we summarize the gene expression changes induced by specific epigenetic modifiers (listed in Table 1), and how they have a variety of intracellular/extracellular consequences to potentiate the effectiveness of subsequent anti-cancer therapies.

**Table 1:** Epigenetic modifiers discussed in this review.

**Type**	**Inhibitor**	**Effect**
DNMTi	Azacitidine	Traps DNMT and prevents its progression along DNA
	Decitabine	Forms a covalent complex with DNMT1 to deplete its activity
	Guadecitabine	-currently unknown-
	Procaine	Prevents the binding of DNMT1 and 3A to DNA
	Zebularine	Traps DNMT and prevents its progression along DNA
HDACi	4-phenylbutyric acid	Pan HDAC inhibitor
	Belinostat	Pan HDAC inhibitor
	Panobinostat	Pan HDAC inhibitor
	Valproic Acid	Pan inhibitor that binds to catalytic center of HDACs
	Dacinostat	Non-direct pan HDAC inhibitor
	Entinostat	Class 1 HDAC inhibitor
	Givinostat	Class 1 and 2 HDAC inhibitor
	Mocetinostat	Inhibits HDAC 1/2/3/11
	Trichostatin A	Inhibits HDACs 1/3/4/6/10
	Vorinostat	Chelator of zinc ions at active sites of HDACs 1/2/4
	Curcumin	Variable; potent effects of HDAC 1/3/8
	Quercetin	-currently unknown-
HDMi	HCI-2509	Inhibits lysine-specific demethylase 1 (LSD1)
	Iadademstat	Inhibits LSD1
	Pargyline	Inhibits LSD1
	S2101	Inhibits LSD1
	SP2509	Inhibits LSD1
	MC3324	Inhibits LSD1 and lysine-specific demethylase 6A
	DW14800	Inhibits protein arginine N-methyltransferase 5 (PRMT5)
	JIB-04	Pan inhibitor of Jumanji-domain histone demethylases
	Methylstat	Inhibits lysine-specific demethylase 4B
	SGC-0946	Inhibits disruptor of telomeric silencing 1-like (DOT1L)
Other	AZD5153	Inhibits bromodomain-containing protein 4 (BRD4)
	JQ1	Inhibits BRD4
	Cl-Amidine	Inhibits protein-arginine deiminase type-4
	EPZ-6438	Inhibits enhancer of zeste homolog 2
	MI-463	Inhibits menin (MEN1)
	MI-503	Inhibits MEN1

## Epigenetic Drug-Induced Sensitization Mechanisms

### Disruption of Pro-survival Signaling

Epigenetic alterations during oncogenesis can dysregulate the expression of growth factor receptors (25). Increased expression of these receptors drives the development of therapy resistance due to the over-activation of their downstream pathways such as PI3K/Akt and subsequent inhibition of cell death (26). While targeted therapies against growth factor receptors have been used to mitigate their effects, the use of such therapies is limited by the rapid development of resistance. Using epigenetic modifiers to control the expression of growth factor receptors is a promising alternative. In breast cancer, dacinostat (HDACi) disrupted epidermal growth factor (EGF)-mediated signaling, which is associated with increased metastasis and cell survival. This was achieved by reducing HER2 (human EGF receptor-2) protein expression via two independent epigenetic mechanisms: first by decreasing *HER2* mRNA level independent of alterations in promoter activity and secondly by increasing proteasomal degradation due to dissociation from its chaperone protein HSP90 via enhanced acetylation (27). In breast cancer as well, treatment with lapatinib (HER2/EGFR kinase inhibitor) and entinostat (HDACi) synergistically disrupted Akt signaling and promoted apoptosis (28). Though the mechanism of entinostat and lapatinib synergy is unknown, it is suggested that this effect is due to entinostat inhibiting lapatinib-induced expression of HER3; a HER2 heterodimerizing partner responsible for resistance to HER2 targeted therapies (29).

Hormone-dependent cancers such as breast and prostate respond to anti-hormone therapy by induction of apoptosis (30). Resistance to such therapy is acquired by downregulation of estrogen receptor (ER) or androgen receptor (AR) via epigenetic mechanisms. Thus, epigenetic drugs have been used to induce ER and AR expression to mediate sensitization to endocrine therapy in breast and prostate cancer, respectively (31–33).

Activation of pro-death pathways has been utilized as a therapeutic target to promote apoptosis in malignant cells. The binding of tumor necrosis factor-related apoptosis-inducing ligand (TRAIL) to its receptors death domain containing receptor (DR) triggers pro-death signaling and induces apoptosis via the caspase cascade (34). Reduced receptor expression is frequently observed in cancer cells resistant to this pathway. Vorinostat (HDACi) sensitized breast cancer cells to TRAIL-induced apoptosis by increasing expression of DR5 (35, 36). Treatment with epigenetic drugs can also lead to hyperactivation of pro-death pathways such as the unfolded protein response (UPR) pathway. UPR is activated to protect cells from endoplasmic reticulum-stress mediated cell death (37). However, when the pathway becomes hyperactivated, this response actually leads to the activation of apoptotic pathways, making it a target in cancer cells. Treatment with methylstat (inhibitor of KDM4B, a lysine-specific histone demethylase) dissociated the UPR-activating initiation factor eIF2α and synergized with PI3K inhibition to hyperactivate UPR gene transcription, culminating in apoptosis (38).

Through modulation of the expression of growth factor receptors or augmenting apoptosis-inducing pathways, epigenetic modifiers can disrupt pro-survival signaling in cancer cells as an efficient mechanism of sensitization (Table 2).

**Table 2:** Epigenetic modifier-induced disruption of pro-survival signaling.

**Malignancy**	**Drug(s)**	**Gene/Protein**	**Mechanism**	**References**
Bladder carcinoma	Decitabine	HOXA9	Restores expression	(39)
Glioblastoma	Decitabine	TP53 and CDKN1A	Restores expression	(40)
Glioblastoma	Decitabine	CASP8	Upregulates expression	(41)
Gastric/esophageal adenocarcinoma	Azacitidine	HPP1	Restores expression	(42)
Renal cell carcinoma	Decitabine	RASSF1A	Restores expression	(43)
Renal cell carcinoma	Decitabine or 4-phenylbutyric acid	miR-492	Restores expression	(44)
ALL	Azacitidine	DCK	Restores expression	(45)
ALL	Azacitidine	AhR	Restores expression	(46)
AML	Azacitidine	SHP-1	Increases expression	(47)
Chronic myeloid leukemia	Azacitidine	PRG2	Increases expression	(48)
Cholangiocarcinoma	Guadecitabine	CDKN2A, RASSF1A, SEMA3B	Increases expression	(49)
Hepatocellular carcinoma	Decitabine	SULF1	Restores expression	(50)
Breast cancer	Dacinostat	HER2	Downregulates expression at mRNA and protein level	(27)
Small cell lung carcinoma	Decitabine/Valproic Acid	CASP8	Restores expression	(51)
Small cell lung cancer	Iadademstat	NOTCH1	Restores expression	(52)
Diffuse large B Cell lymphoma	Panobinostat	NOXA	Increases expression	(53)
Prostate	Trichostatin A	ATF3/4	Increases expression	(54)
Prostate	Azacitidine	GST	Restores expression	(55)
Prostate	Azacitidine	miR-34a	Restores expression	(56)
Tongue squamous cell carcinoma	Trichostatin A	miR-375	Increases expression	(57)
Solid tumors	Mocetinostat	miR-203	Restores expression	(58)
Ovarian	Zebularine	RASSF1A, ARHI, BLU	Restores expression	(59)
Bladder	Trichostatin A	CXADR	Increases expression	(60)
Breast	Vorinostat	DR4/DR5	Increases expression	(36)
Breast	Vorinostat	DR5	Increases expression	(35)
T-cell leukemia	HDACi	TRAIL-R2, c-FLIP, and Apaf-1	Increases expression	(61)
Breast	Entinostat	ERα and CYP19A1	Increases expression	(31)
Breast	Vorinostat	ERα	Increases expression	(32)
Prostate	Quercetin/Curcumin	AR	Increases expression	(33)
ALL	Vorinostat	BCR-ABL	Decreases expression	(62)
T-cell ALL	Dacinostat	c-FLIP	Decreases expression along with increasing DR4/5 expression to sensitize to Apo2L/TRAIL-induced apoptosis	(63)
Chronic myeloid leukemia	Dacinostat	BCR-ABL	Decreases expression	(64)
AML	Dacinostat	FLT-3	Decreases expression and activity	(65)
Mixed lineage leukemia	MI-463/MI-503	HOXA9	Decreases expression	(66)
MLL	Azacitidine	TERT	Decreases expression	(67)
Hepatocellular carcinoma	Guadecitabine	WNT/EFG/IGF	Decreases expression of pathway associated genes	(68)
Non-small cell lung carcinoma	Panobinostat	TAZ	Decreases transcription and its targets (EGFR and EGFR ligand)	(69)
Multiple myeloma	EPZ-5676/SGC-0946	IRF4	Decreases expression	(70)
Hematologic	Vorinostat	JAK	Decreases expression	(71)
Breast	Entinostat	Akt	Inhibits phosphorylation	(28)
Breast	Methylstat	eIFα	Increases dissociation from KDM4B leading to increased phosphorylation by ERK and transcription of unfolded protein response genes	(38)
Breast	MC3324	ERα	Inhibits signaling	(72)
Breast	Cl-amidine	Akt/mTOR	Inhibits signaling, leading to increased nuclear accumulation of p53	(73)
Colon	Decitabine	Akt	Inhibits signaling	(74)
Colon	Cl-amidine	p53	Increases transcription of targets, including miR-16	(75)
Retinoblastoma	Vorinostat	NFκB	Inhibits signaling and increases p53 expression	(76)
Gynecologic	Panobinostat	Mutant TP53	Decreases protein expression	(77)
Gynecologic	SP2509	p62	Stabilizes protein expression	(78)
AML	Panobinostat	Akt/NFκB	Inhibits signaling to increase p53-mediated cell death	(79)
Non-small cell lung carcinoma	Panobinostat	EGFR	Inhibits signaling	(80)
Ovarian	S2101	Akt	Inhibits phosphorylation	(81)
Prostate	Azacitidine	Akt	Inhibits activation	(82)

### Restoration of Cell Cycle Control and Disruption of DNA Damage Repair

Cancer cells often rely on a dysregulated cell cycle for their continued proliferation (83). Epigenetic modifiers can restore tight control of the cell cycle and proliferation by mediating a reversal of dysregulated gene expression as a mechanism to potentiate therapy. Entinostat (HDACi) downregulated the expression of MYC, E2F, and other G2M cell cycle genes to sensitize breast cancer cells to doxorubicin-induced growth arrest, however, how these genes are downregulated is unknown (84). Previously, Lee et al. showed entinostat treatment in breast cancer inhibited Akt signaling (28). Since Akt signaling controls cell cycle (85), it is likely that Akt is involved in entinostat-mediated doxorubicin sensitization. While entinostat alone inhibited the expression of cell cycle proteins, its combination with decitabine (DNMTi) in pancreatic cancer increased expression of p21 to reinstitute cell cycle control and inhibit tumor growth, likely due to increased acetylation of histone H3 and demethylation of the p21 promoter (86).

Epigenetic modifiers can potentially mitigate the effects of fusion oncoproteins. Gene fusions formed as a result of chromosomal translocations are often responsible for oncogenesis and therapy resistance (87, 88). In Ewing sarcoma, the EWS/Fli1 fusion gene is a key oncogenic driver. Treatment with JIB-04 (pan inhibitor of Jumanji-domain histone demethylases) simultaneously increased expression of cell-cycle inhibitor genes while suppressing expression of proteins that promote cell cycle, possibly through a disruption of EWS/Fli1 fusion gene program (89).

Hyperactive DNA damage repair pathways in cancer cells promote resistance to DNA damaging chemotherapeutics and radiation (90). In neuroblastoma, treatment with vorinostat (HDACi) diminished the expression of Ku-86, a key protein in non-homologous end joining DNA damage repair, to potentiate the anti-neoplastic effects of DNA damaging radiation (91). How vorinostat affects Ku-86 expression requires further study. Expression of DNA damage repair proteins like 53BP1 and RAD51 was also downregulated following treatment with pevonedistat (NEDD8-activating enzyme inhibitor) and belinostat (HDACi) in acute myeloid leukemia (AML). Downregulation of these proteins occurred in response to pevonedistat-mediated inhibition of belinostat-induced NFκB signaling and belinostat-mediated inhibition of pevonedistat-induced Chk1/Wee1 signaling, identifying a reliance of the two drugs on each other to disrupt DNA damage repair (92).

Restoring control of cell cycle progression and diminishing the activation of DNA damage repair pathways is a promising mechanism to improve responses to treatment. Epigenetic modifiers offer a unique route to achieving this objective (Table 3).

**Table 3:** Restoration of cell cycle control and disruption of DNA damage repair by epigenetic modifiers.

**Malignancy**	**Drug(s)**	**Target Gene/Protein**	**Mechanism**	**References**
Breast	Cl-amidine	CDKN1A and GADD45A	Increases expression to inhibit cell cycle	(93)
Breast	Decitabine/ Trichostatin A	MSH2	Restores expression	(94)
Colorectal	AZD5153	c-Myc/Wee1	Reduces expression	(95)
Gastric	Procaine	CDKN2A and RARβ	Restores expression	(96)
Acute leukemia	Decitabine	CDKN2A	Restores expression	(97)
Non-small cell lung carcinoma	Trichostatin A	CDKN1A	Increases expression to mediate G1 arrest	(98)
Non-small cell lung carcinoma	Azacitidine	MGMT	Restores expression	(99)
Diffuse large B cell lymphoma	Decitabine	SMAD1	Restores expression	(100)
MDS/Chronic myeloid leukemia	Decitabine	CDKN2B	Restores expression	(101)
Multiple myeloma	Azacitidine/EPZ-6438	SMAD3	Restores expression	(102)
Ovarian/Colon	Decitabine	MLH1	Restores expression	(103)
Pancreatic	Decitabine/Vorinostat	CDKN1A	Increases expression to mediate G1 arrest	(86)
Pancreatic	Azacitidine	SST and SSTR2	Restores expression	(104)
Multiple	HDACi	SLFN1	Restores expression	(105)
Solid tumors	Azacitidine/HDACi	Genes related to ionizing radiation	Increases expression for radiosensitivity	(106)
Bladder	Panobinostat	MRE11	Reduces expression to increase radiosensitivity	(107)
Neuroblastoma	Vorinostat	Ku-86	Reduces expression to disrupt DNA damage repair	(91)
Neuroblastoma	Panobinostat	Chk1	Reduces expression and signaling to disrupt DNA damage repair	(108)
Breast	Entinostat	MYC, E2F, and G2M cell cycle genes	Reduces expression to induce G2M cell cycle arrest	(84)
Non-small cell lung carcinoma	Belinostat	ERCC1	Decreases expression to disrupt DNA damage repair	(109)
Lung adenocarcinoma	HCI-2509	PLK1	Decreases expression and target genes	(110)
Non-Hodgkin's Lymphoma	Belinostat	c-Myc	Decreases expression to increase DNA damage	(111)
Ovarian	Panobinostat	RAD51	Decreases expression to increase PARP inhibiton	(112)
Pancreatic	JQ1	c-Myc	Decreases expression	(113)
Testicular	Guadecitabine	p53	Increases activation and target gene expression	(114)
Breast	Valproic Acid	γH2AX and H3S10p	Increases and decreases retention, respectively	(115)
Breast/Ovarian	Guadecitabine	PARP	Increases “trapping” by PARP inhibitors	(116)
Ewing Sarcoma	JIB-04		Disrupts EWS/Fli1 oncogeneic program to increase DNA damage	(89)
AML	Belinostat	Chk1/Wee1	Inhibits signaling to disrupt DNA damage response	(92)
Chronic myeloid leukemia	Decitabine/Vorinostat	p53	Increases cell death through p53-dependent pathway and p21	(117)
AML	Azacitidine/Panobinostat	p53 signaling	Induced remission in patient-derived xenograft models	(118)
AML	Panobinostat	Chk1/Wee1	Decreases signaling to disrupt DNA damage response	(119)
AML	Trichostatin A	γH2A.X	Accumulates to enhance radiosensitivity	(120)
Non-small cell lung carcinoma	Decitabine/Trichostatin A	miRNAs	Enhances DNA damage by dysregulating expression	(121)
Non-small cell lung carcinoma	Panobinostat	p53/p21 and Chk1	Increases expression of p53-dependent pathway and decreased Chk1 signaling	(122)
Ovarian	Guadecitabine	DNA repair genes	Alters expression to disrupt DNA damage repair	(123, 124)
Solid tumors	DNMTi/HDACi		Reduces chromatin condensation to increase DNA damage following chemotherapy	(125)

### Suppress Immune Evasion/Augmenting Immune Responses

The immune system plays a pleiotropic role in cancer progression. Infiltration of immune cells into the tumor microenvironment releases a plethora of cytokines and growth factors that contribute to tumor proliferation, survival, and metastasis. Concurrently, activation of immune cells to target cancer is a promising strategy to utilize the host immune system in the fight against cancer (126). Like other anti-cancer treatments, malignant cells develop a resistance to immunotherapies by evading or suppressing the immune system and its activation via aberrant epigenetics (127, 128). Treatment with epigenetic modifiers has proved successful in augmenting immunotherapy. For a detailed review on this topic, please refer to Gomez et al. (129).

Epigenetic modifiers trigger increased expression of proteins for targeted therapies including immunotherapies. Trichostatin A (HDACi) up-regulated the mRNA and protein levels of both MIC-A and ULBP-2 in glioblastoma, which are recognized by natural killer (NK) cells to increase NK cell-mediated lysis (130). Entinostat (HDACi) blocked regulatory T cells (which negatively regulate the immune system and limit the efficacy of immunotherapy) in renal cell carcinoma via increased STAT3 acetylation, possibly due to increased CBP/p300 expression that acetylates STAT3 (131). In ovarian and colon cancer, azacitidine (DNMTi) increased the expression of multiple cancer cell-specific antigens. Since these antigens can be recognized by the host immune system, they represent prime targets for immunotherapies (132). The increased expression of cancer antigens also provides ample opportunity for the development of anti-cancer vaccines directing the host immune system to target these antigens. Such advances are currently in their infancy but have the potential for exceptional breakthrough in cancer treatment, especially when combined with epigenetic modifiers.

In non-small cell lung cancer, azacitidine (DNMTi) and givinostat (HDACi) induced Type I interferon signaling through transcriptional downregulation of MYC to increase the expression of the T cell chemoattractant CCL5, thereby reversing tumor immune evasion by promoting T cell infiltration. This combination also shifted host T cells from exhausted states (characterized by loss of effector function due to prolonged antigen stimulation) to memory and effector states [capable of durable responses to immune checkpoint blockades) via activation of associated genes (133)].

Cancer cells exploit the “immune checkpoint” function to evade the immune system (134) by expression of programmed cell death-1 (PD-1) or anti–cytotoxic T-lymphocyte-associated antigen-4 (CTLA-4) resulting in increased apoptosis of T cells. Immune checkpoint blockers such as nivolumab (monoclonal antibody blocking PD-1) and ipilimumab (monoclonal antibody blocking CTLA-4) have emerged as an attractive mechanism to decrease immune system evasion and tumor cell survival. Co-administration of azacitidine (DNMTi) and entinostat (HDACi) alongside immune checkpoint blockers improved treatment outcome in a preclinical metastatic cancer model via their inhibitory action on the myeloid derived suppressor cells within the tumor microenvironment (135).

Exploitation of the immune system to successfully diminish tumor burden is a promising avenue of improving anti-cancer therapy. The use of epigenetic modifiers offers a distinctive method to potentiate these therapies (Table 4).

**Table 4:** Suppression of immune evasion/augmented immune responses following epigenetic modifier treatment.

**Malignancy**	**Drug(s)**	**Gene/Protein**	**Mechanism**	**References**
Osteosarcoma	Entinostat	MIC-A and MIC-B	Increases expression to increase NK cell-mediated cytotoxicity	(136)
Glioblastoma	Trichostatin A	MIC-A and ULBP-2	Increases expression to increase NK cell-mediated death	(130)
Colon	Decitabine/Vorinostat	Fas	Increases expression to sensitize to FasL-induced apoptosis and improve CTL adoptive transfer immunotherapy	(137)
Melanoma	Vorinostat	DR5	Increases expression to overcome immune resistance	(138)
Melanoma	Dacinostat	MHC and tumor antigen	Increases expression to improve functional activity of lymphocytes	(139)
Renal cell carcinoma /Prostate	Entinostat	STAT3	Increases acetylation to improve immunotherapy	(131)
Non-small cell lung carcinoma	Azacitidine/Givinostat	MYC	Inhibits signaling to reverse immune evasion	(133)
Ovarian	Azacitidine	Type I interferon	Activates signaling to reduce immunosuppression	(140)
Colon/Ovarian	Azacitidine	Cancer antigens	Vaccines	(132)
AML	Azacitidine	PD-1, PD-L1, and CTLA-4	Nivolumab and Ipilimumab	NCT02397720

### Modulation of Microenvironmental Interactions

Cellular and extracellular matrix interactions within the tumor microenvironment are crucial for cancer development and progression. Epigenetic dysregulation in cancer is known to control adhesion through a variety of mechanisms (141–143). Thus, the use of epigenetic modifiers could provide a way to mollify these alterations. In solid tumors, disengagement from the microenvironment has severe consequences for the patient, as it is the first step in metastasis (144). Therefore, increasing cell adhesion proves beneficial to localize the tumor to the primary site.

In a majority of solid tumors, carcinomas arise from epithelial cells undergoing epithelial to mesenchymal transition (EMT), which causes loss of epithelial polarity/adhesion and increased migratory/invasiveness potential (145). Following EMT, cancer cells acquire stem-cell like properties and a higher rate of metastasis (146). EMT is controlled by multiple epigenetic mechanisms, including DNA methylation and histone modifications (147). The expression of the classical cell adhesion molecule and EMT suppressor E-cadherin is downregulated via promoter hypermethylation in cancer cells (148), or repressed by transcription factor Snail (149) in conjunction with histone modifiers such as lysine-specific histone demethylase 1 (LSD1) recruited by Snail (150). In breast cancer cells, EMT was suppressed by the LSD1 inhibitor pargyline (151). It is important to note, that the same study identified LSD1 to inhibit M1 macrophage infiltration into tumors, which is known to promote tumor progression and therapy resistance (152).

Targeting the SNAIL/LSD1 complex to prevent EMT via depletion of SNAIL expression was accomplished by the BRD4 inhibitor JQ1 in breast cancer. JQ1 repressed the expression of Gli1, an important mediator of *SNAIL* transcription. This prevented SNAIL-mediated repression of epithelial markers such as E-cadherin and prevented EMT (150, 153). Combined, these two studies provide a powerful indication of how the use of epigenetic modifiers can perturb EMT to prevent metastasis and improve treatment efficacy in solid tumors.

In hematologic malignancies, interactions within the bone marrow microenvironment transition malignant cells into chemoresistant states (154). Disruption of these interactions mobilizes cells from the bone marrow into the peripheral blood, thereby sensitizing them to therapy. In acute lymphoblastic leukemia (ALL), azacitidine (DNMTi) and panobinostat (HDACi) combined to disrupt cellular adhesion within the bone marrow microenvironment in ALL by decreasing the surface expression of the tetraspanin protein CD81, resulting in increased chemosensitivity (155, 156).

Hypoxia within the tumor microenvironment can often promote therapy resistance (157). This therapy resistance can be attributed to multiple factors including aberrant micro RNA (miRNA) expression and dysregulated epigenetic machinery (158, 159). Thus, gene expression alterations are accumulated and therapy resistance can occur in a variety of mechanisms such as those described in Figure 1. Due to the aberrant epigenetics involved, the use of epigenetic modifiers could sensitize cancer cells by reverting these hypoxic effects. However, further study is required to elucidate their effectiveness.

The role of microenvironmental interactions and their effect on cancer progression has been well-defined, however, the use of epigenetic modifiers to attenuate these effects has not been exploited. More studies across all cancer subtypes are necessary to achieve a greater understanding of how microenvironmental interactions can be modulated by epigenetic therapy.

### Reprogramming of Cellular Metabolism

Through a variety of genetic and epigenetic mechanisms, metabolic reprogramming can render cancer cells resistant to chemotherapeutics (160–162). These changes can often result as a compensatory mechanism in response to the exposure of certain chemotherapeutics (162). Therefore, targeting aberrant cellular metabolism is a promising method of circumventing therapy resistance.

Due to the epigenetic regulation involved with aberrant metabolism, epigenetic modifiers could prove highly successful in mitigating the resultant chemoresistant effects. Treatment with entinostat (HDACi) combined with cisplatin upregulated the gene expression of thioredoxin-interacting protein (TXNIP), which inhibited the cellular uptake of glucose and increased DNA damage (163). This occurred via an increase in *TXNIP* promoter activity, however, this increase was only achievable with the two drugs in combination. In AML, treatment with the DNMTi azacitidine combined with the Bcl-2 inhibitor venetoclax disrupted cellular metabolism by decreasing glutathione levels, thereby diminishing electron transport chain complex II activity and oxidative phosphorylation (164).

Epigenetic modifiers can also augment the effectiveness of established antimetabolites like pemetrexed, which targets enzymes like thymidylate synthase (TI) catalyzing purine and pyrimidine synthesis. TI expression can be augmented post treatment with pemetrexed, thus leading to resistance (165). In non-small cell lung cancer, pemetrexed treatment followed by givinostat (HDACi) downregulated the mRNA and protein expression of TI, thereby overcoming therapy resistance and resulting in a synergistic increase in cell death (166).

While there has been strong evidence of the role played by epigenetic-induced metabolic changes in cancer cells in promoting therapy resistance, the study of how epigenetic modifiers can mitigate these effects has yet to be explored in depth. More examination into these effects is required in order to better overcome resistance to therapies.

### Opportunities for Development of Rational Combinations With Epigenetic Therapy

The impact of epigenetic modifiers on global gene expression results in modulation of several genes, both promoting and inhibiting therapy resistance, thereby necessitating and offering opportunity to combine with targeted therapies. This is exemplified by a study in ovarian cancer that identified overexpression of CD146, a cell surface marker involved in tumor dissemination, following treatment with vorinostat (HDACi). This increased expression was exploited by combining vorinostat with anti-CD146 monoclonal antibody treatment to synergistically induce cell death via inhibition of CD146-mediated Akt signaling (167). Vorinostat (HDACi) along with decitabine (DNMTi) was also observed to significantly increase the expression of the tyrosine kinase AXL in AML. This led to the identification of a novel triple therapy with the AXL inhibitor BGB324 facilitating synergistic activation of cell death (168). Therefore, mechanistic understanding of epigenetic drug action is essential for developing rational combinations with targeted therapies.

## Need for Clinical Trials

The use of epigenetic modifiers is a robust method for improving treatment efficacy in cancer. Through a variety of mechanisms, epigenetic therapy has the potential to augment the effectiveness of cancer treatments to improve overall survival in patients. In many of the examples presented above, a combination of epigenetic modifiers was used to induce specific changes that potentiate the effects of anti-cancer therapeutics in cancer cells. However, despite a plethora of clinical trials involving the use of epigenetic modifiers, very few have focused on the use of a combination of epigenetic modifiers along with anti-cancer therapy (Table 5). The combination therapies identified in this review underline the need and provide the basis for the development of future clinical trials to study their effectiveness.

**Table 5:** List of clinical trials utilizing multiple epigenetic modifiers in combination with traditional therapy.

**Malignancy**	**Epigenetic Modifiers**	**Other Therapeutics**	**NCT Identifier**
ALL	Decitabine/Vorinostat	Vincristine/Dexamethasone/Mitoxantrone/Pegasparagase/Methotrexate	01483690
AML	Azacitidine/Vorinostat	Gemtuzumab	00895934
AML	Azacitidine/Valproic Acid	All-trans retinoic acid/Hydroxyurea	01369368
AML/MDS	Azacitidine/Valproic Acid	All-trans retinoic acid	00339196
Breast	Decitabine/Panobinostat	Tamoxifen	01194908
Lymphoma	Azacitidine/Vorinostat	Gemcitabine/Busulfan/Melphalan/Dexamethasone/Caphosol/Glutamine/Pyridoxine/Rituximab	01983969
MDS	Azacitidine/Valproic Acid	All-trans retinoic acid	00326170
MDS	Decitabine/Vorinostat	CD3-/CD19- NK cell infusion	01593670
MDS	Azacitidine/Valproic Acid	All-trans retinoic acid	00439673
Melanoma	Decitabine/Panobinostat	Temozolomide	00925132
Non-small cell lung cancer	Azacitidine/Entinostat	Docetaxel/Gemcitabine/Irinotecan	01935947
Non-small cell lung cancer	Azacitidine/Entinostat	Nivolumab	01928576

Additionally, it is worth mentioning that not only the use of epigenetic modifiers (either alone or in combination with one another) in conjunction with chemotherapeutics should be studied, but the protocols in which they are administered should be considered as well. Simultaneous exposures have been shown to have an inhibitory effect on cell viability compared to sequential treatment (169). Additionally, a study of the use of azacitidine and panobinostat in B-ALL identified that following treatment in mice; leukemic cells were mobilized from the bone marrow into the peripheral blood. This mobilization was responsible for the improved efficacy of subsequent chemotherapy treatment, thus suggesting that staggering the treatments had a significant effect (170). More study on this effect as well as its potential in other cancer subtypes must be performed to exploit the efficacy of epigenetic treatments.

## Conclusions

Aberrant epigenetics is responsible for the development and progression of several cancers. These alterations can be the driving forces of therapy resistance and survival. Treatment with epigenetic modifiers offers a unique route to diminishing these effects and re-sensitizing cancer cells to traditional therapies. While there have been some clinical trials studying the efficacy of epigenetic modifiers in cancer, more studies focusing on identifying specific gene targets are required, particularly with a combination of epigenetic modifiers in conjunction with other therapies. By precisely identifying sensitization biomarkers, epigenetic/chemotherapeutic/immunotherapeutic combination therapies can achieve greater translational success (171). Follow-up studies using comprehensive analyses like RNAseq, global methylation, and chromatin immunoprecipitation-Seq are required to identify pathways of sensitization.

It is also imperative to include analyses of non-coding regions of the DNA, such as miRNA. While epigenetic alterations during oncogenesis directly affect the transcription of coding genes, these variations can have an effect on the expression of miRNAs (172, 173), which are non-coding RNAs that function in RNA silencing and post-transcriptional regulation of gene expression. miRNAs can mediate either tumor suppressive or oncogenic effects depending on their gene target (174). Examination of alterations in miRNA expression following treatment with epigenetic modifiers could identify additional sensitization mechanisms and therapeutic markers.

Studies investigating the development of inhibitors of atypical histone modifications, such as citrullination, phosphorylation, sumoylation, ubiquitylation, and ribosylation; are needed because these modifications are also known to regulate gene transcription and contribute to cancer progression (175–178). The mechanisms outlined in this review offer not only a rationale for successful combinations and mechanisms, but also identify indications for their use in specific patients based on the markers being modulated, in line with the advancements in personalized medicine. Further studies on the mechanisms of epigenetic modifier action in cancer are needed to identify markers that can detect and predict clinical response.

## Author Contributions

AQ wrote the draft manuscript and generated figures and tables. AG and SB edited the manuscript. All authors approved the final version of the manuscript.

## Conflict of Interest

The authors declare that the research was conducted in the absence of any commercial or financial relationships that could be construed as a potential conflict of interest.
